# Clinical Decision Support for Antibiotic Prescribing Duration in Children With Acute Otitis Media

**DOI:** 10.1001/jamanetworkopen.2025.60066

**Published:** 2026-02-18

**Authors:** Matt Mason, Sara Ruth Slovin, Arezoo Zomorrodi, Shannon Chan, Rebecca Judge, Jennifer Vodzak, Craig Shapiro

**Affiliations:** 1 Department of Pharmacy, Nemours Children’s Hospital Delaware, Wilmington; 2 Clinical Informatics, Nemours Children’s Hospital Delaware, Wilmington; 3 Department of Emergency Medicine, Nemours Children’s Hospital Delaware, Wilmington; 4 Division of Infectious Diseases, Nemours Children’s Hospital Delaware, Wilmington; 5 Division of Infectious Diseases, Children’s National Hospital, Washington, DC

## Abstract

This quality improvement study evaluates whether use of an antibiotic order panel is associated with increased proportion of prescriptions for acute otitis media with guideline-concordant durations.

## Introduction

Acute otitis media (AOM) is the most common indication for antibiotics in children.^1^ The American Academy of Pediatrics recommends 10 days of therapy for children younger than 2 years and shorter durations for those 2 years or older.^2^ Despite evidence showing no difference in outcomes with shorter durations in older children, many patients still receive 10 days of therapy.^3^ Clinical decision support (CDS) embedded in the electronic medical record (EMR) may optimize prescribing practices.^4,5^ We aimed to assess CDS’s role in improving antibiotic durations in children with AOM.

## Methods

This single-center, retrospective quality improvement study analyzed prescriptions written during primary care or emergency department (ED) visits for AOM. The Nemours Children's Health Institutional Review Board deemed the study exempt from review and informed consent because it was not human participant research. We followed the SQUIRE reporting guideline.

Antibiotic order panels for amoxicillin and amoxicillin-clavulanate were developed by the antimicrobial stewardship program in collaboration with clinical informatics. The panels defaulted to the recommended dose, frequency, and duration based on indication and age. Clinicians had the ability to modify any of the defaulted fields. Order panels for amoxicillin and amoxicillin-clavulanate were implemented in September 2021 and March 2022, respectively. The study period included prescriptions from September 2021 to November 2023. The primary outcome was the proportion of prescriptions written for guideline-concordant durations: 10 days for children younger than 2 years and 5 to 7 days for children 2 years or older.

Patient demographics, diagnosis, and prescription data were extracted from the central EMR. The diagnosis grouper search term was *acute otitis media*, including *International Statistical Classification of Diseases and Related Health Problems, Tenth Revision* diagnosis codes H65 to H75; visits for recurrent AOM were included. Logistic regression models were developed to assess implications of the order panel and other confounding variables for the primary outcome.

Two-sided *P* < .05 indicated statistical significance. All data processing and statistical analysis were performed from September 2021 to November 2023 using Python 3.13.5 (Python Software Foundation).

## Results

There were 26 382 unique prescriptions for amoxicillin and amoxicillin-clavulanate included for analysis. Of these prescriptions, 41.9% and 58.1% were written for children younger than 2 years and 2 years or older, respectively. Patients had a median (IQR) age of 2 (1-4) years and included 14 141 males (53.6%).

Order panel use was associated with a significantly higher proportion of prescriptions written for the recommended duration (90.2% vs 56.1%; difference, 34.1 [95% CI, 32.9-35.2] percentage points; *P* < .001) (Table). The outcome was most pronounced in children 2 years or older (85.2% vs 26.3%; *P* < .001) (Figure).

**Table.** Proportion of Prescriptions Written for Guideline-Concordant Duration of Therapy

Variable	Prescriptions, No. (%)		Difference (95% CI), percentage points	*P* value
	Order panel use (n = 9322)	No order panel use (n = 17 060)		
Total population	8408 (90.2)	9575 (56.1)	34.1 (32.9-35.2)	<.001
Age group, y				
0-1	3657 (97.6)	7005 (95.8)	1.8 (1.1-2.4)	<.001
≥2	4751 (85.2)	2570 (26.4)	58.8 (56.9-60.9)	<.001
Visit type				
Office	6331 (88.5)	7480 (52.2)	36.3 (34.9-37.6)	<.001
Emergency department	2077 (95.9)	2095 (76.6)	19.3 (17.3-21.3)	<.001
Antibiotic				
Amoxicillin	6539 (92.4)	8002 (56.5)	35.9 (34.7-37.1)	<.001
Amoxicillin-clavulanate	1869 (83.2)	1573 (54.1)	29.1 (26.1-32.1)	<.001

**Figure.**
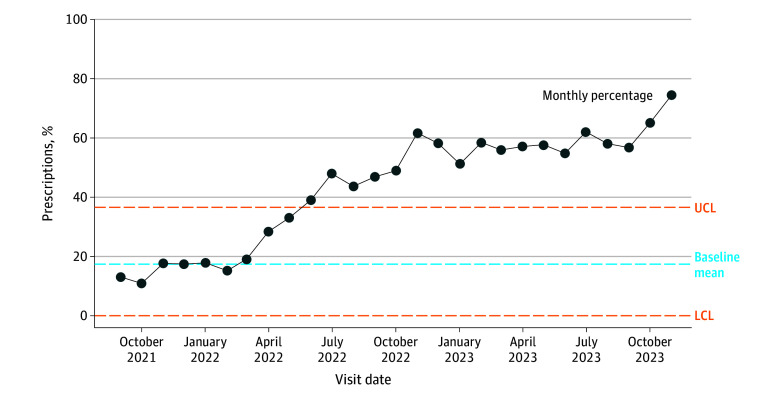
Statistical Process Control Chart of Prescriptions Written With Guideline-Concordant Duration of Therapy in Children 2 Years or Younger Baseline monthly mean of guideline-concordant durations of therapy was determined using the previous 24 months of therapy prior to order panel implementation (ie, September 2019-September 2021). LCL indicates lower control limit, and UCL indicates upper control limit.

Univariate logistic regression showed order panel use was associated with higher odds of guideline-concordant durations (odds ratio [OR], 16.1; 95% CI, 14.8-17.6; *P* < .001). Multiple logistic regression identified age, race, encounter type, and panel use as significant variables. ED encounters were associated with an increase in the proportion of guideline-concordant durations, while prescribing by nonphysician clinicians (ie, advanced practice professionals) and amoxicillin-clavulanate use were associated with a decrease. Of the significant variables identified, order panel use was associated with the highest odds of guideline-concordant durations (OR, 20.0; 95% CI, 18.2-22.0; *P* < .001).

## Discussion

The findings corroborate reports from other studies that leveraged the EMR to improve antibiotic stewardship.^6^ The intervention was effective across both office and ED visits. A large proportion of prescriptions from the order panel were modified to a longer therapy duration immediately after implementation. Education was provided to reinforce the order panel’s default to the guideline-concordant duration, which was associated with an increase in the primary outcome over time. Study limitations include the lack of clinical outcomes, inability to exclude recurrent infections or concomitant diagnoses, and absence of clinician-level data.

Antibiotic order panels embedded in the EMR were associated with increased guideline-concordant durations of therapy for AOM. CDS, combined with education and stakeholder engagement, is a powerful tool for antimicrobial stewardship. As contemporary evidence supports shorter therapy durations, implementation of low-cost, easily scalable interventions can assist with decreasing antibiotic exposure for AOM.

## Notes

Supplement.
Data Sharing Statement
