# Supplementary material for: Clinical Decision Support for Antibiotic Prescribing Duration in Children With Acute Otitis Media
Source: JAMA Netw Open. 2026 Feb 18;9(2):e2560066. doi: 10.1001/jamanetworkopen.2025.60066 (PMC12917673; doi:10.1001/jamanetworkopen.2025.60066)
Supplement: Supplement. — Data Sharing Statement [file jamanetwopen-e2560066-s001.pdf]

Data Sharing Statement

Mason. Clinical Decision Support for Antibiotic Prescribing Duration in Children With Acute Otitis Media. *JAMA Netw Open*. Published February 18, 2026.
doi:10.1001/jamanetworkopen.2025.60066

Data
Data available: Yes
Data types: Data dictionary
How to access data: I can provide the a data dictionary upon request via encrypted email at mathew.mason@nemours.org
When available: With publication

Supporting Documents
Document types: Statistical/analytic code
How to access documents: I can post the Python code on github.com
When available: With publication

Additional Information
Who can access the data: Researchers whose proposed use of the data has been approved
Types of analyses: Data processing and cleaning, statistical analysis, data visualization, and machine learning
Mechanisms of data availability: Signed data access agreement
